# Retraction: Inventorization of traditional ethnobotanical uses of wild plants of Dawarian and Ratti Gali areas of District Neelum, Azad Jammu and Kashmir Pakistan

**DOI:** 10.1371/journal.pone.0286781

**Published:** 2023-06-14

**Authors:** The PLOS ONE Editors

The *PLOS ONE* Editors retract this article [1] because it was identified as one of a series of submissions for which we have concerns about authorship, competing interests, and peer review. We regret that the issues were not addressed prior to the article’s publication.

MI did not agree with the retraction. All other authors either did not respond directly or could not be reached.
